# Antiviral RNAi Response in *Culex quinquefasciatus*-Derived HSU Cells

**DOI:** 10.3390/v15020436

**Published:** 2023-02-04

**Authors:** Mine Altinli, Mayke Leggewie, Jonny Schulze, Rashwita Gyanwali, Marlis Badusche, Vattipally B. Sreenu, Janina Fuss, Esther Schnettler

**Affiliations:** 1 Bernhard-Nocht-Institute for Tropical Medicine, 20359 Hamburg, Germany; 2 German Center for Infection Research, Partner Site Hamburg-Lübeck-Borstel-Riems, 20359 Hamburg, Germany; 3 MRC-University of Glasgow-Center for Virus Research, Glasgow G61 1QH, UK; 4 Institute of Clinical Molecular Biology (IKMB), Kiel University, 24105 Kiel, Germany; 5 Faculty of Mathematics, Informatics and Natural Sciences, Universität Hamburg, 20148 Hamburg, Germany

**Keywords:** RNA interference, *Culex quinquefasciatus*, *Aedes aegypti*, RNAi, antiviral immunity

## Abstract

*Culex* spp. mosquitoes are important vectors of viruses, such as West Nile virus, Eastern equine encephalitis virus and Rift valley fever virus. However, their interactions with innate antiviral immunity, especially RNA interference (RNAi), are not well known. Most research on RNAi pathways in mosquitoes is focused on the tropical vector mosquito *Aedes aegypti*. Here, we investigated the production of arbovirus-specific small RNAs in *Cx. quinquefasciatus*-derived HSU cells. Furthermore, by silencing RNAi-related proteins, we investigated the antiviral role of these proteins for two different arboviruses: Semliki Forest virus (SFV) and Bunyamwera orthobunyavirus (BUNV). Our results showed an expansion of Ago2 and Piwi6 in *Cx. quinquefasciatus* compared to *Ae. aegypti*. While silencing Ago2a and Ago2b increased BUNV replication, only Ago2b showed antiviral activity against SFV. Our results suggest differences in the function of *Cx. quinquefasciatus* and *Ae. aegypti* RNAi proteins and highlight the virus-specific function of these proteins in *Cx. quinquefasciatus*.

## 1. Introduction

Mosquitoes are efficient vectors for many arthropod-borne viruses (arboviruses). Arboviruses are maintained in nature by replicating in both their vertebrate hosts and mosquito vectors. During replication in mosquitoes, arboviruses must overcome the antiviral defence mechanism, called RNA interference (RNAi). RNAi is a sequence-specific RNA degradation pathway subdivided into several pathways, depending on the involvement of different specific key proteins and the characteristics of the produced small RNA molecules [1,2,3,4,5,6,7,8].

These RNAi pathways have been extensively studied in the model organism *Drosophila melanogaster*, and the results have often been extrapolated to mosquitoes. Over the last few years, research on *Aedes aegypti* has increased and has revealed a more individualised picture with both similarities and differences to the model organism [1,2,3,4,5,6]. For instance, the exogenous small interfering (exo-si)RNA pathway is considered to be the main antiviral defence mechanism for both *D. melanogaster* and mosquitoes [7,8]. The siRNA pathway involves the production of double-stranded small RNAs, which are 21 nts in length, by the protein Dicer-2 (Dcr2). These siRNAs are incorporated into a multiprotein complex (RNA-inducing silencing complex), where they specifically associate with Argonaute-2 (Ago2). Once associated, one of the siRNA strands is used as a guide strand to bind complementary sequences in the cell and initiate their degradation [7]. Notably, the production of viral-derived (v)siRNAs during infection, as well as the antiviral properties of Ago2 and Dcr2, has been confirmed for a large collection of arboviruses; however, this has mostly been tested in *Ae. aegypti* [8]. 

In contrast, the PIWI-interacting small (pi)RNA pathway is mainly associated with transposon control in *D. melanogaster* and other insects but is also thought to have an antiviral role in *Ae. aegypti* mosquitoes. The expansion of the family of piRNA-related genes encoded by *Ae. aegypti* compared to drosophila supports additional functions of the piRNA pathway [2,3,5]. Evidence for the piRNA pathway’s antiviral role has been found in the form of viral-derived (v)piRNA molecules in infected mosquitoes [9,10,11,12,13,14,15,16,17,18,19]. In *Ae. aegypti*, mature vpiRNAs are produced through the ping-pong amplification cycle, a production loop that depends on Ago3 and the Piwi protein Piwi5 (and to a lesser extent, Piwi6) [12,18,19,20]. Mature piRNAs are 24–30 nts in length and are characterised by a U1 antisense or A10 sense sequence bias (uridine at the 1st or adenine at the 10th position). Although not directly involved in the production of piRNAs, the silencing of *Ae. aegypti* Piwi4 transcripts resulted in increased virus infection for several tested arboviruses [11,13,14,15,18,21,22]. 

Many mosquito-borne viruses of medical and veterinary importance are transmitted by mosquitoes of *Aedes* ssp. (e.g., *Ae. aegypti* and *Ae. albopictus*). However, mosquitoes of the genus *Culex* are also relevant vectors, as they are involved in the transmission of arboviruses such as West Nile virus (WNV), Japanese encephalitis virus (JEV), Venezuelan equine encephalitis virus (VEEV) and Rift valley fever virus (RVFV) [23]. Nevertheless, existing knowledge about antiviral RNAi in mosquitoes is based nearly exclusively on *Ae. aegypti*. Although it is tempting to extrapolate this knowledge to other mosquito species, differences in the number and homologues of Argonaut and Piwi family proteins between mosquito genera hint towards a more complex picture [3]. Characterising the RNAi defence mechanism in all vector species could provide targets for new vector or virus control measures.

In this study, we established an in vitro silencing protocol for transcripts of key RNAi proteins in *Cx. quinquefasciatus*-derived HSU cells to study the antiviral RNAi response against two arboviruses from different families: the Bunyamwera orthobunya virus (BUNV; Orthobunyavirus, *Peribunyavirida*e) and Semliki Forest virus (SFV; Alphavirus, *Togaviridae*). Together with small RNA sequencing analysis, this allows us to compare these results with previous results observed in *Ae. aegypti*-derived cells. The patterns of produced BUNV-specific small RNAs in infected HSU and *Ae. aegypti*-derived cells look similar, showing the production of vsiRNA and vpiRNA-like small RNAs. Regarding the antiviral activity of key RNAi proteins, differences were observed between SFV and BUNV. 

## 2. Materials and Methods

### 2.1. Cell Lines and Mosquitoes

HSU cells are derived from *Cx. quinquefasciatus* ovarian tissue [24]. The cell line was maintained at 28 °C in Leibovitz-15 medium (Thermo Fisher Scientific Inc., Waltham, MA, USA) supplemented with 10% foetal bovine serum (FBS; ThermoFisher Scientific), 10% tryptose phosphate broth (TPB; Gibco Life Technologies) and antibiotics (penicillin–streptomycin; ThermoFisher Scientific). The baby hamster kidney cell line (BHK-21) and BSRT7 cells (BHK-21 cells stably expressing T7 RNA polymerase) were maintained at 37 °C and 5 % CO_2_ in Glasgow Minimum Essential Medium (GMEM; ThermoFisher Scientific) supplemented with 5% FBS (ThermoFisher Scientific), 10% TPB (Thermo Fisher Scientific ) and antibiotics (penicillin–streptomycin; ThermoFisher Scientific). 

### 2.2. Viruses

SFV6-2SG-NLuc [25], BUNV and BUNV-NLuc [13] were produced and titred as previously described. The recombinant viruses were produced by transfecting the corresponding plasmids (CMV-SFV6-2SG-NLuc or pT7riboBUNL(+), TVT7BUNM-NL and pT7riboBUNS(+), respectively) into either BHK or BSRT7 cells. Upon observation of the cytopathic effect (CPE), the supernatant was collected and used for virus stock production. The virus stocks (of all three viruses) were produced by inoculating BHK-21 cells in supplemented GMEM medium and incubating them at 37 °C. The virus was harvested upon the detection of the CPE. 

The virus titre was determined via a plaque assay on BHK-21 cells using an overlay consisting of 1.2% Avicell (FMC biopolymer) and 2 × MEM supplemented with 4% FBS in a 1:1 mixture. Briefly, 3 × 10^5^ BHK-21 cells per well were seeded in a 12-well plate and kept at 37 °C overnight. Subsequently, the cells were infected with a serial dilution of the virus stock prepared in GMEM medium and incubated at 37 °C for 1 h. Each dilution step was performed in duplicate. Then, a 2 mL overlay was added per well. Cells infected with SFV6-NLuc, BUNV or BUNV-NLuc were fixed with 4% formaldehyde-PBS at 48 or 72 h post-infection (hpi) and stained with crystal violet solution.

### 2.3. dsRNA Design and Synthesis

*Cx. quinquefasciatus* RNAi protein sequences were searched using blastp based on their homology to previously annotated *Ae. aegypti* RNAi proteins (Table S1). We aligned the sequences [26] and build a phylogenetic tree using PhyML [27]. We used the naming of *Cx. quinquefasciatus* RNAi proteins based on their similarity to *Ae. aegypti* proteins, which slightly differs from the previous literature [2,28]. Primers specific to *Cx. quinquefasciatus* RNAi transcripts were designed to amplify at least 120bp nucleotide stretches that are unique to a given transcript. cDNA was produced using oligodT primers (Thermo Fisher Scientific) and M-MLV (Promega, Fitchburg, WI, USA) from total RNA isolates of HSU cells. The expression of the transcripts in HSU cells was determined using the above-produced cDNA and transcript-specific primers, followed by Sanger sequencing of the produced PCR product. 

Target-specific primers flanked by the T7 RNA polymerase promotor sequence (Table S2) were used to amplify the unique target-specific sequence from HSU-derived cDNA. Upon validation by Sanger sequencing, the PCR products were used for in vitro transcription using the MEGAscript RNAi kit (Thermo Fisher Scientific). 

### 2.4. Growth Kinetics

A total of 5 × 10^4^ HSU cells per well were seeded in a 96-well plate and kept at 28 °C overnight. Cells were infected either with BUNV-NLuc at a multiplicity of infection (MOI) of 0.1 or with SFV-Nluc at an MOI of 10 or MOI of 0.1. The virus inoculum was replaced with fresh medium after 1–2 h of incubation. Infection was performed in duplicate and in at least two independent experiments.

Cells were subsequently sampled at 1, 2 and 3 days post-infection. For this, the medium was removed from the well and replaced with the passive lysis buffer (50 µL/well, Promega). After an incubation period of 30 min at room temperature, the lysate was transferred to an Eppendorf tube and stored at −20 °C until the analysis using the NanoGlo Luciferase assay kit (Promega).

### 2.5. Small RNA Sequencing and Analysis

A total of 10^6^ HSU cells per well were seeded in a 6-well plate in duplicate, infected with BUNV (MOI 0.1) and incubated at 28 °C for 48 h. Total RNA was isolated using Trizol (Thermo Fisher Scientific Inc., Waltham, MA, USA), and Glycogen was used as a carrier during isopropanol precipitation, according to the manufacturer’s instructions. Total RNA (100 ng) was used for library preparation with the NEXTFLEX^®^ Small RNA-Seq Kit v3 (PerkinElmer Inc., Waltham, MA, USA) according to the manufacturer’s protocol. Subsequently, Illumina-based small RNA sequencing of 18–35 nt long RNAs on one lane NovaSeq6000 SP v1.0 (2 × 50 bp) at IKMB (Kiel, Germany) was performed as previously described [25]. For the analysis of small RNAs (sRNAs), tools and resources available with the RNA Workbench 2.0 [29] were used. First, the reads were mapped against the respective reference genome using BWA. Uniquely mapped reads were sorted into three separate libraries. The complete set of all 18–31 nt long reads were counted and plotted against their respective sizes. All 26–29 nt long reads were included in the creation of a nucleotide versus cycle plot to show nucleotide position biases and were used to compute sequence overlaps using signature.py [30]. These and all 21 nt long reads were used to create bed graphs of the genome coverage distribution of reads per base. The accession numbers are NC_001927.1; NC_001926.1; and NC_001925.1. Raw small RNA data can be found at the NCBI Sequence Read Archive with biosample accession codes SAMN31680994 and SAMN31680995.

### 2.6. Knockdown and Infection Experiments

A total of 2.5 × 10^5^ HSU cells per well were seeded in a 24-well plate and kept at 28 °C overnight. Cells were then transfected with 200 ng of transcript-specific dsRNA or control dsRNA (eGFP-specific) using Dharmafect2 reagent (Horizon Discovery Ltd., Cambridge, UK). 

To confirm the knockdown efficiency, the RNA of cells was isolated at 24 h post-transfection (hpt) using Trizol (Thermo Fisher Scientific). Technical duplicates were combined for RNA isolation, and RNA was isolated according to the manufacturer’s instructions. Then, 2.5 µg of total RNA was used to produce cDNA with M-MLV reverse transcriptase (Promega) and Oligo(dT)15 primers (Thermo Fisher Scientific) according to the manufacturer’s instructions. 

SYBR Green PCR using transcript-specific primers (Table S2) and GADPH as a housekeeper transcript was performed. The relative amount of target mRNA was calculated using the 2^−ΔΔCT^ method with eGFP dsRNA samples as a control. qPCR reactions were performed at least in technical duplicates.

To determine the effects on virus infection upon knockdown, the cells were again transfected with 200 ng of sequence-specific dsRNA or eGFP control dsRNA per well using Dharmafect2 (Horizon Discovery Ltd.). Twenty-four hours post-transfection, cells were infected with BUNV-NLuc (MOI 0.1) or SFV6-2SG-NLuc (MOI 10) and subsequently allowed to incubate at 28 °C. Forty-eight hours post-infection, the medium was removed from the cells and replaced with passive lysis buffer (100 µL/well; Promega). After an incubation period of 30 min at room temperature, the lysate was further processed using the NanoGlo Luciferase assay kit (Promega) according to the manufacturer’s instructions. Luminescence was subsequently detected using a GloMax luminometer (Promega). Experiments were performed in duplicate and independently repeated at least three times. 

### 2.7. BUNV Viral DNA Detection

A total of 2.5 × 10^5^ HSU cells per well were seeded in a 24 well-plate and either infected the following day with BUNV at an MOI of 2 or mock-infected. After 1–2 h incubation at 28 °C, the cell medium was replaced with fresh medium. The supernatant and cells were collected at 2 and 6 dpi. The supernatant was used to check successful BUNV infection and amplification (and the lack of BUNV infection in mock-infected cells), performing a TCID50 on BHK cells. DNA was isolated from cell pellets using the QIAamp DNA isolation kit (QIAGEN, Hilden, Germany) following the manufacturer’s protocol. Isolated DNA was treated with RNAase A for 1h at 37 °C (AppliChem, Darmstadt, Germany). Subsequently, PCR was performed using the DNA samples as the template with GoTaq polymerase (Promega) and BUNV S segment-specific primers (Table S3). Due to the observation of faint bands, PCR reactions were repeated using the same primers and the produced PCR product as a template. Following gel purification, the PCR product was sequenced with Sanger sequencing (LGC genomics, Hoddesdon, UK). 

### 2.8. Statistical Analysis

For all other statistical analyses, R version 4.1.2 (1 November 2021) was used [31]. Data were first tested for normality using the Shapiro–Wilk test. For data that are not normally distributed, the Kruskal–Wallis test (stats package), followed by Dunn’s test (dunn.test package [32]), was used with Bonferroni corrections for multiple comparisons. *p* < 0.05 was considered statistically significant. 

## 3. Results

### 3.1. BUNV and SFV Growth Kinetics in HSU Cells

**Figure 1:**
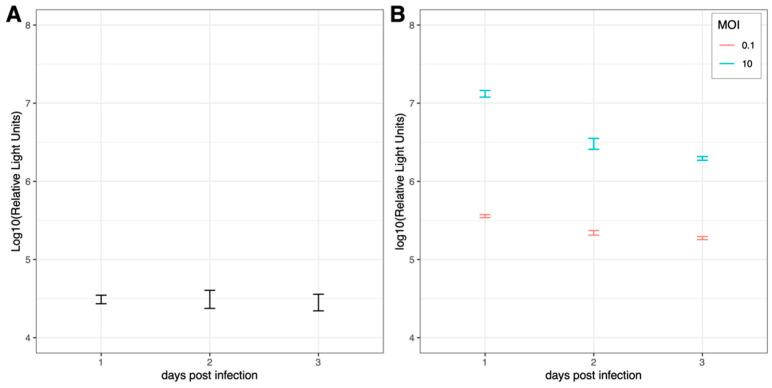
BUNV (**A**) and SFV (**B**) growth kinetics in *Cx. quinquefasciatus*-derived HSU cells. HSU cells were inoculated either with BUNV-NLuc (MOI 0.1) or with SFV-NLuc (MOI 0.1 or MOI 10) expressing NLuc as a reporter protein. The mean of three independent experiments performed in technical duplicates is shown with standard error.

To investigate the growth kinetics of BUNV and SFV in HSU cells, cells were inoculated either with BUNV-NLuc (MOI 0.1) or with SFV6-Nluc (MOI 10 or MOI 0.1), both expressing NLuc as a reporter protein. Samples were collected daily for three days. BUNV-NLuc was stably translated in HSU cells over three days post-infection (Figure 1A). In contrast, a decrease in Nluc (Figure 1B) or virus titre (Figure S1) was observed over time in SFV-infected HSU cells. Initial Nluc expression proved the ability of SFV to infect and translate viral proteins in HSU cells (Figure 1B). 

### 3.2. BUNV-Specific Small RNAs in Cx. quinquefascitus-Derived HSU Cells

**Figure 2:**
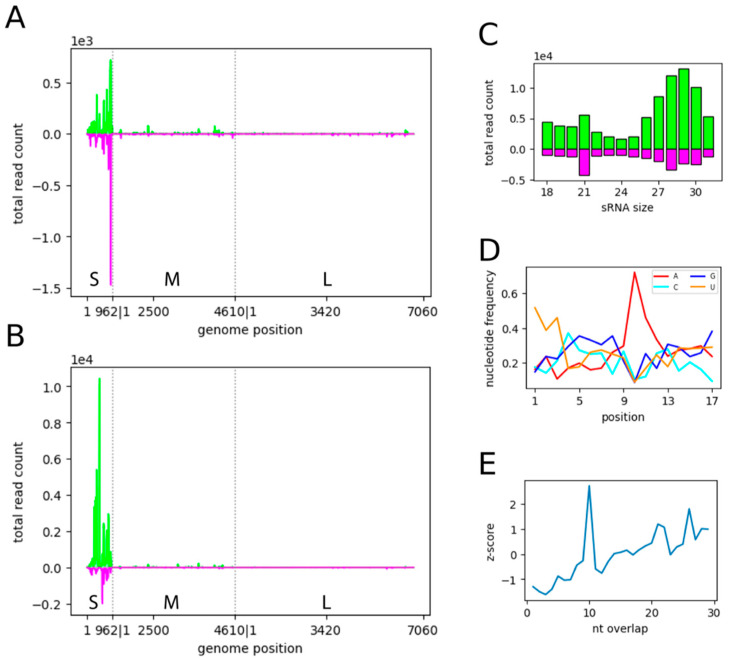
Small RNA analyses of BUNV-infected *Cx. quinquefasciatus*-derived HSU cells**.** Small RNAs of HSU cells were mapped to the BUNV genome and antigenome. (**A**) Distribution of the 21 nt small RNAs or (**B**) 26–29 nt small RNAs along the genome and antigenome of the three segments of BUNV (S, M, L). (**C**) Length distribution of BUNV-specific small RNAs. Positive numbers are RNAs mapping to the antigenome of BUNV (green)*,* while negative numbers indicate RNAs mapping to the genome of BUNV (pink). Y-axis: absolute count of small RNAs. (**D**) Relative nucleotide frequency and conservation per position of 26–29 nt small RNAs mapping to the BUNV genome or antigenome. (**E**) The overlap z-score indicating the probability of overlap between the genome and antigenome of 26–29 nt BUNV-specific small RNAs was calculated. Two independent experiments were carried out, and the results of one representative experiment are shown here (Figure S2).

Due to the low virus production of SFV in infected HSU cells (even at MOI 10; Figure S1), the production of virus-specific small RNAs in infected HSU cells was only performed for BUNV; where an increase in virus production over time was observed (Figure S1). Cells were infected with BUNV (MOI 0.1), and small RNA was sequenced and analysed at 48 hpi. BUNV-specific small RNAs of 21 nt in length were identified for all three segments (Figure 2C and Figure S2). The S and M segments showed several distinct areas of vsiRNA production across the viral sequence, mapping to the genome and antigenome in similar amounts; however other areas showed no or very low amounts of vsiRNA production (Figure 2A and Figure S2). In contrast, the vsiRNAs mapping to the L segment were clustered in the first and last part of the genome, with the middle part producing little to no vsiRNA (Figure 2A and Figure S2).

Similar to vsiRNAs, small RNAs in the size range of piRNAs (26–29 nts) were identified for all three segments (Figure 2B and Figure S2). These vpiRNA-sized small RNAs showed the specific characteristics linked to the ping-pong production pathway of piRNAs, including A10 and U1 bias (Figure 2D and Figure S2) and the 10 nt overlap of sense and antisense small RNAs (Figure 2E and Figure S2). Overall, more vpiRNA-like molecules were produced than vsiRNAs for all three segments (Supplementary Table S4). Comparing the three segments, the S segment was the main producer of both vsiRNA and vpiRNA-like small RNAs (Figure 2A,B). The hot spots where high amounts of small RNAs along the BUNV genome were produced were similar for vsiRNA and vpiRNA-like molecules. 

**Figure 3:**
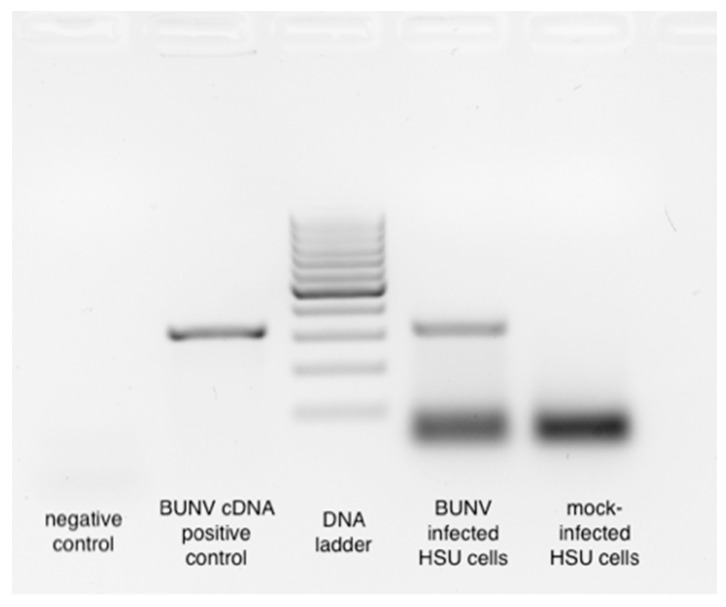
BUNV-specific viral (v)DNA production in infected *Cx. quinquefasciatus*-derived HSU cells. DNA of BUNV- and mock-infected HSU cells were collected at 6 dpi. PCR using primer set 3 (Table S3) was performed. cDNA of BUNV-infected cells was used as a positive control.

In *Ae. aegypti* and derived cells, evidence suggests that, generally, vpiRNA production is dependent on the production and presence of viral-derived DNA (vDNA) [15]. To determine whether BUNV infection resulted in vDNA production in HSU cells, we designed four primer sets spanning the BUNV S-segment, as most BUNV-specific piRNA-like small RNAs map to this region (Figure 2B). PCR was performed using isolated DNA from BUNV- or mock-infected HSU cells at 2 or 6 dpi. Mock-infected cells did not show any bands for any of the primer pairs or time points (Figure 3, data not shown). In contrast, BUNV-infected HSU cells showed a BUNV-specific band at 6 dpi (Figure 3) for one primer set (402–724 nt antigenome). The sequencing of the PCR product confirmed its BUNV specificity. The other three primer sets showed no specific PCR band, although the positive control (cDNA of BUNV-infected cells) resulted in PCR products, and TCID50s performed on the supernatants of the infected HSU cells (Figure S1A) ensured successful BUNV infection and replication. The results suggested that vDNA was produced in the later stages of infection. The lack of PCR products for the mock-infected DNA of HSU cells (Figure 3, data not shown) did not support the presence of BUNV-like endogenous viral elements (EVEs) in the genome of HSU cells, which could induce the production of BUNV-specific piRNAs.

### 3.3. Effects of Silencing of RNAi Transcripts on Arbovirus Infection

Using the recently updated *Cx. quinquefasciatus* genome (annotated version), sequences of key RNAi-related proteins of *Ae. aegypti* were used to search for homologous protein sequences (blastp) in the *Cx. quinquefasciatus* genome. As previously described [2,28], *Cx. quinquefasciatus* encodes nearly all known *Ago* and *Piwi* genes found in *Ae. aegypti*. Here, we named the proteins according to their similarity to *Ae. aegypti* proteins (Figure S3). We designed primers for dsRNA production and checked for the expression of the RNAi-related transcripts in our HSU cells. Due to the high overlap of the nucleotide sequences of Piwi1 and Piwi3 transcripts, dsRNA was designed to knock down the expression of both (indicated as Piwi1/3). Furthermore, the expression of Piwi7 was very low and, therefore, was excluded from further experiments. Following the verification of the presence of all other transcripts in our HSU cells, their antiviral activity was investigated. 

**Figure 4:**
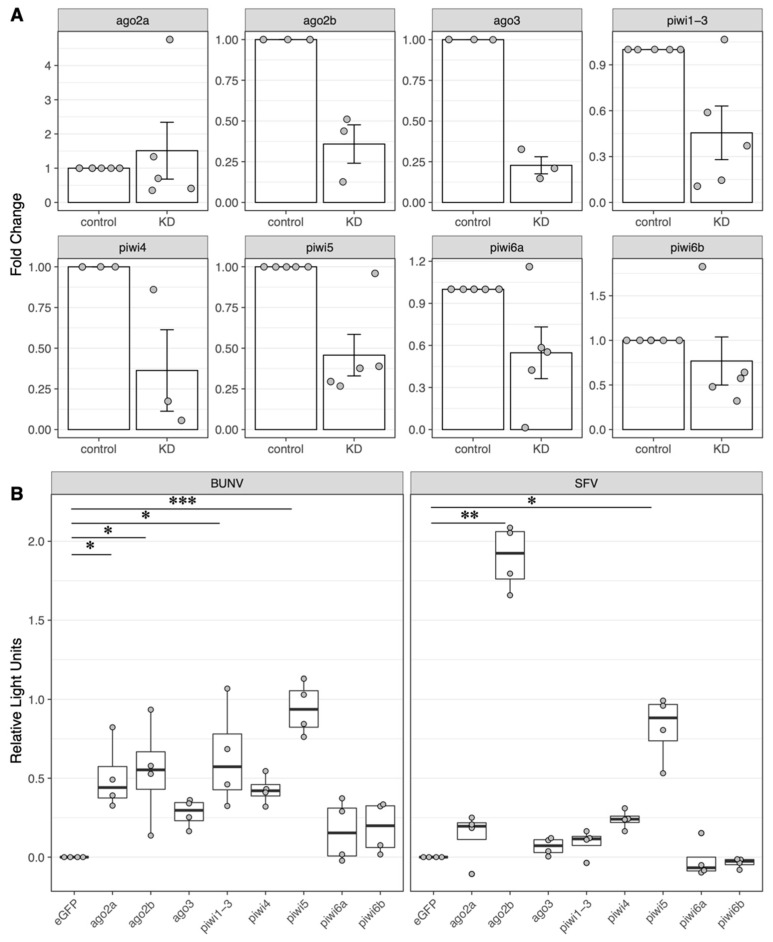
Silencing of RNAi transcripts and its effect on arbovirus infection in HSU cells. HSU cells were transfected either with gene-specific dsRNAs (Ago2a, Ago2b, Ago3, Piwi1/3, Piwi4, Piwi5, Piwi6a, Piwi6b) or with control dsRNA (eGFP-specific). The following day, cells were infected with BUNV-NLuc (MOI 0.1) or SFV-NLuc (MOI 10). To confirm the silencing of the transcripts (**A**), mRNA targets were quantified using gene-specific primers and GADPH mRNA as a housekeeper transcript. Fold change in mRNA targets was calculated using the 2^−ΔΔCT^ method with eGFP dsRNA samples as the control. The mean of at least 3–5 replicates and the standard error of the mean are shown. To investigate the effect on arbovirus infection (**B**), Nanoluciferase activity was measured and normalised to samples transfected with eGFP dsRNA as a control (*: *p* < 0.05, **: *p* < 0.001, ***: *p* < 0.01). Boxplots show the median of 4 independent experiments performed in technical duplicates.

To determine the antiviral activity of these RNAi-related proteins against both BUNV and SFV, dsRNA-based silencing experiments were performed. For most of the transcripts, dsRNA-based silencing was efficient on average over the independent replicates (ago2b, ago3, piwi1/3, piwi 4, piwi 5, piwi 6a), while for some of them (ago2a, piwi 6b), silencing was less efficient and varied more in different replicates (Figure 4). 

BUNV infection increased when the cells were transfected with dsRNA against Piwi5, Ago2a, Ago2b and Piwi1/3 compared to the eGFP control. The strongest increase was detected for Piwi5. While silencing Ago3 and Piwi4 caused an increase in BUNV infection, this increase was not statistically significant. While the growth curves showed that SFV replication in HSU is inefficient, silencing Ago2b or Piwi5 increased SFV infection significantly, with the strongest effect observed for Ago2b. The silencing of any other RNAi-related transcripts had no effect on SFV infection in HSU cells. 

## 4. Discussion

The interaction of arboviruses with the antiviral RNAi response in mosquitoes is mostly studied in *Ae. aegypti* [10,12,13,14,15,18,19,22,33,34,35], and although *Culex sp.* mosquitoes are also important arbovirus vectors, their RNAi response is much less known. Here, we studied the antiviral activity of *Cx. quinquefasciatus* RNAi-related proteins, linked to the siRNA or piRNA pathway, against two arboviruses from different families: Semliki Forest virus (*Togaviridae*) and Bunyamwera orthobunyavirus (*Peribunyaviridae*).

Rückert et al. looked at the induction of piRNA-related transcripts in *Cx. quinquefasciatus* upon WNV infection [28]. However, no induction was observed, similar to observations for siRNA-pathway-related transcripts in *Ae. aegypti* [2]. dsRNA-based silencing approaches have identified several key RNAi proteins in *Ae. aegypti* that show antiviral activity against all, or at least most, tested viruses [36]. The expansion of Ago2 in *Cx. quinquefasciatus*, known as a key protein in the antiviral siRNA pathway, suggests the presence of different complexes in the siRNA pathway of *Cx. quinquefasciatus*. Another possibility could be the dysfunctionality of one Ago2 homologue. However, both *Culex* homologues harbour the amino acids known to be important for the catalytic slicing activity of Ago2 [2]. In addition, silencing experiments showed antiviral activity against BUNV for both homologues. Interestingly, only one homologue (Ago2b) showed antiviral activity against SFV. This suggests some virus specificity of the Ago2 homologues. More research with additional viruses is needed to identify any possible mechanisms behind this virus specificity.

In *Ae. aegypti*, Piwi4 has been shown to possess antiviral activity against all tested arboviruses, including BUNV and SFV [11,13,15,18,21,22]. In contrast, no increase in SFV or BUNV infection was observed upon the silencing of Piwi4 in HSU cells. Interaction studies in *Ae. aegypti-*derived cells showed the binding of Piwi4 with key proteins of the siRNA and piRNA pathways [18]. Moreover, Piwi4 has recently been reported to exert antiviral activity by binding vpiRNAs, specifically ones derived from viral (v)DNA [15]. vDNA is produced in mosquitoes and derived cells upon infection with RNA arboviruses. Its production and presence have been linked to the establishment of persistent arbovirus infection [15,37]. A possible explanation for the lack of Piwi4 antiviral activity could be that *Cx. quinquefasciatus* or specifically HSU cells lack such a vDNA production response. However, the production of WNV-specific vDNA in infected HSU cells has been reported, in general, suggesting the presence of such a pathway in HSU cells [28]. More importantly, BUNV-specific vDNA was detected in infected HSU cells. 

In *Ae. aegypti*, Piwi5 and Ago3 are the main producers of vpiRNAs, and silencing them results in the reduction in/lack of the corresponding vpiRNAs [10,12,19,22]. However, no increase in virus replication has been reported upon Piwi5 or Ago3 silencing in *Ae. aegypti*-derived cells yet [10,11,12,13,14,19,22]. In contrast, the silencing of Piwi5 in HSU cells resulted in an increase in virus infection for both tested viruses. At the moment, it is not known whether Piwi5 of *Cx. quinquefascitus* is involved in the production of vpiRNAs, similar to Piwi5 in *Ae. aegypti*. Small RNA sequencing of BUNV-infected HSU cells has shown the ability of HSU cells to produce vsiRNA and vpiRNA-like small RNAs, similar to the ones previously reported in *Ae. aegypti*-derived cells [13]. This again substantiates that HSU cells have active siRNA and piRNA pathways, and both pathways can be induced by an acute arbovirus infection. Therefore, the previously reported lack of WNV-specific piRNAs in acutely infected HSU cells might be a virus-specific observation [28]. Until now, vpiRNA production in HSU cells had only been reported for the persistently infecting mosquito-specific Merida virus (MERDV) [28]. In contrast to MERDV-derived piRNA-like small RNAs that target very specific small areas of the genome, BUNV-specific vpiRNA-like small RNAs target a higher proportion of the genome, although still in defined regions. Whether or not this is linked to the different viruses or the difference in infection status (persistent versus acute) is not known yet.

Previous studies in *Ae. aegypti* suggested that vpiRNA production in infected cells is dependent on either the production of vDNA or the presence of EVEs with similar nucleotide sequences [15]. BUNV-specific piRNA-like small RNAs were mostly mapped across the whole S-segment. However, BUNV-specific vDNA was only detected for an approximately 230 nt long stretch of the S-segment. In addition, no PCR bands were observed in non-infected HSU cells, suggesting that EVEs are not involved in the production of BUNV-specific piRNAs like small RNAs in HSU cells. 

It has been previously proposed that distinct Piwi proteins are involved in various piRNAs’ biogenesis depending on the substrate and corresponding piRNAs. Transposon-specific piRNAs depend on Piwi4, 5 and 6 and Ago3, and piRNA biogenesis from different alphaviruses (SINV, SFV and CHIKV) predominantly requires Piwi5 and Ago3 only, while flavivirus (DENV) piRNA biogenesis requires Piwi6 [10,12,19]. An expansion in the *Piwi6* gene family (*Piwi6a* and *Piwi6b*) is observed in *Cx. quinquefasciatus* compared to *Ae. aegypti*. It is possible this expansion represents further specialisation for the production of piRNAs from different RNA sources, such as RNA substrates from different viruses or the recognition of different replication strategies. However, at the moment, it is not known whether *Cx. quinquefasciatus* Piwi6 has a similar function or whether Piwi6a and Piwi6b vary in their biological activity in *Cx. quinquefasciatus*. Nevertheless, both transcripts could be detected in HSU cells, but neither of them showed antiviral activity against the tested viruses under the tested conditions. 

Piwi1, (2) and 3 of both *Ae. aegypti* and *Cx. quinquefasciatus* are mostly expressed in germline cells, and research on their biological function is very limited [21,38]. Hence, it is difficult to assess the biological significance of the lack of a distinct Piwi2 homologue in *Cx. quinquefasciatus* compared to *Ae. aegypti*. In addition, Piwi1 and Piwi3 transcripts show high nucleotide sequence homology, making it difficult to silence them individually by sequence-specific dsRNAs to study their function separately. However, the silencing of Piwi1/3 increased BUNV replication in HSU cells, suggesting an antiviral role for these proteins. 

Follow-up experiments in *Cx. quinquefasciatus* mosquitoes are needed to determine whether all presented results from *Cx. quinquefasciatus-*derived HSU cells can be transferred. However, previous research findings, for example, for *Ae. aegypti*, strongly support cell lines as valid models to investigate the interaction of viruses with the RNAi response in mosquitoes. 

## 5. Conclusions

Our knowledge of the antiviral RNAi response in *Culex* sp. mosquitoes is limited, although such knowledge of the complex interactions of arboviruses with their mosquito vectors is important to understand restriction factors for arbovirus replication in mosquitoes.

*Cx. quinquefasciatus* expresses homologues for most of the known key *Ae. aegypti* RNAi proteins, with additional expansion in Piwi6 and Ago2 homologues. The acute arbovirus infection of *Cx. quinquefasciatus*-derived HSU cells with BUNV induces the production of vsiRNA and vpiRNA-like molecules. 

The silencing of RNAi transcripts, followed by BUNV or SFV infection in derived cells, has suggested several antiviral proteins, with only some shared between *Ae. aegypti* and *Cx. quinquefasciatus*. Comparing the antiviral activity in *Cx. quinquefasciatus-*derived HSU cells between SFV and BUNV also highlights differences in the antiviral response against arboviruses belonging to different arbovirus families. 

## Data Availability

The RNA sequencing data generated here will be available in the NCBI Sequence Read Archive (biosample accession codes: SAMN31680994, SAMN31680995). The qRTPCR and luciferase data will be made available upon request.

## Supplementary Materials

The following are available online at https://www.mdpi.com/article/10.3390/v15020436/s1. Table S1. VectorBase gene identifiers of RNAi protein transcripts identified in *Culex quinquefasciatus* and corresponding orthologues in *Aedes aegypti*; Table S2**.** Primers used for dsRNA production (T7 primers) and relative quantification (qPCR primers); Table S3**.** Primers used for vDNA detection; Table S4**:** Number of small RNA-seq data (total raw reads, BUNV-specific, 21 BUNV-specific, 26–29 BUNV-specific) from both repeats. Figure S1**:** BUNV-Nluc and SFV-Nluc growth kinetics in HSU cells. Figure S2**:** small RNA-seq data from BUNV in HSU cells (second repeat). Figure S3: *Cx. quinquefasciatus* and *Aedes aegypti* RNAi proteins.
