# Supplementary material for: Antiviral RNAi Response in Culex quinquefasciatus-Derived HSU Cells
Source: Viruses. 2023 Feb 4;15(2):436. doi: 10.3390/v15020436 (PMC9968050; doi:10.3390/v15020436)
Supplement: Supplementary file 1 [file viruses-15-00436-s001.zip › viruses-2069972-supplementary.pdf]

Supplementary data, Altinli et al.

Table S1. VectorBase gene identifiers of RNAi transcripts identified in *Culex quinquefasciatus* and corresponding orthologues in *Aedes aegypti*.

Transcript Name	Gene identifier *Ae. aegypti*	Gene identifier *Cx. quinquefasciatus*
Argonaute-1 (Ago1)	AAEL012410	XM_038258517.1, XM_038258518.1, XM_038258516.1, XM_038258515.1, XM_038258522.1, XM_038258521.1, XM_038258519.
Argonaute-2a (Ago2a)	AAEL017251	XM_038263062.1/2
Argonaute-2b (Ago2b)	AAEL017251	XM_038262260.1
Argonaute-3 (Ago3)	AAEL007823	XM_038254040.1
Piwi1	AAEL007823	XM_001844015.2
Piwi2	AAEL008098	
Piwi3	AAEL013692	XM_038255983.1
Piwi4	AAEL007698	XM_038251629.1
Piwi5	AAEL013233	XM_038251859.1
Piwi6A	AAEL013227	XM_038251858.1 (XM_038251857.1)
Piwi6B	AAEL013227	XM_038258148.1
Piwi7	AAEL006287	XM_038266499.1

Table S2. Primers used for dsRNA production (T7 primers) and relative quantification (qPCR primer) for the respective target RNAi transcripts. Primers are presented in the 5′→3′ direction. The T7 RNA polymerase promotor sequence is highlighted in italics.

Name	Accession Number	Primers with *T7* (5′-3′), FW/RV	qPCR Primer (5′-3′) FW/RV
Ago2a	XM_038263062.1/2	*TAATACGACTCACTATAGGG* CCGCTAAACCGAAAGGAAAC/ *TAATACGACTCACTATAGGG* ACTTTTGCTGCTTCTGCTG	CAGATGGACAAGGTTGGGG T/CAGTTTGGAGCCAAAGAC CAC
Ago2b	XM_038262260.1	*GTA ATA CGA CTC ACT ATA GGG* GGCGGTTCTCAGCAGTATGG/ *GTA ATA CGA CTC ACT ATA GGG* TCTTGCTGCTCCTGTTGCTT	CACAAGTCCGGGCGTGAA/ GGGGGAAGATTCTGGTTAC GG
Ago3	XM_038254040.1	*GTA ATA CGA CTC ACT ATA GGG* GGCCTTCATCGTGGTTCAGA/ *GTA ATA CGA CTC ACT ATA GGG* CGGGCCAGTTGTAGTACAGG	CTGTCAGTACGCCCACAAG A/ TATCGTTCAGCACCTCGTCC
Piwi1	XM_001844015.2	*TAATACGACTCACTATAGGG* ATCGGGGACACTCTTCGAAC/ *TAATACGACTCACTATAGGG* CACACGAGAATGTCCTGCTC	GGGGTATGCGGTTCGAAAT C/ ACCGATCCGCTTTGTTGTTC
Piwi3	XM_038255983.1	*TAATACGACTCACTATAGGG* ATCGGGGACACTCTTCGAAC/	GGGGTATGCGGTTCGAAAT C/ ACCGATCCGCTTTGTTGTTC

		*TAATACGACTCACTATAGGG*CACACGAG AATGTCCTGCTC	
Piwi4	XM_038251629.1	*GTA ATA CGA CTC ACT ATA GGG* CGGCTAGTTGAGGTTCGAGG/GTA *ATA CGA CTC ACT ATA GGG* CGATCGCCCAATGTTTGAGC	ACGCCCGAAAATGTCTGAC C/GTCACGACCCTCACTGCT AT
Piwi5	XM_038251859.1	*TAATACGACTCACTATAGGG* CACTACCAAGCTGAGCATGC/*TAATACG ACTCACTATAGGG* GTGCCAACCTTACGCAACTT	ACGAGACACAGACTTCGCA G/CCATCTCCGTCGTTCCATC A
Piwi6a	XM_038251858.1 (XM_038251857.1)	*TAATACGACTCACTATAGGG* CCGACGCAGGTAATCAAGTG/*TAATACG ACTCACTATAGGG*CAATCTTGTCCCTGAT GGCG	CTACATTACCAGCATCCGA CAG/TGCACTTCTCAAACAG GTCG
Piwi6b	XM_038258148.1	*TAATACGACTCACTATAGGG* CGGAGGTTATCAACATGGCG/*TAATACG ACTCACTATAGGG* TCGCACAGCTTGTTCCTAGA	TCAAGGTGCTCATGGAATC G/GACCGTTGAGTAGAATTC CGAG

Table S3. Primers used to detect viral DNA production.

Primer Name	Sequence (5′-3′), FW/RV	Amplicon Size
BUNV-set1-F	ACTCCACACTACAAACTTGC/ ACTGGGTTGTTCCGGTTG	330 bp
BUNV-set2-F	CCTCTCCATCATTCCAAGTG	290 bp
BUNV-set3-F	AGATGCTGAAAGTCAGTGAACC/ CCAACCTAAGCTAGAGACAAC	322 bp
BUNV-set4-F	ACAATGCGGCAGAGGTAC/ TTAGCCCGCTGTCTTTCTG	286 bp

Table S4. Small RNA-seq data from both repeats.

	Total Raw Reads	Mapped Reads (BUNV-Specific)	% Total	Total siRNA (21 nt)	%Mapped siRNA	Total piRNA (26–29 nt)	%Mapped piRNA	siRNA/piRNA
E-518 (Figure 3)	26,773,368	1,545,163	0.058	205,590	0.133	1,339,573	0.867	0.15
G-520 (Supplementary Figure S1)	24,362,700	29,464,450	1.209	557,655	0.019	28,906,795	0.981	0.02

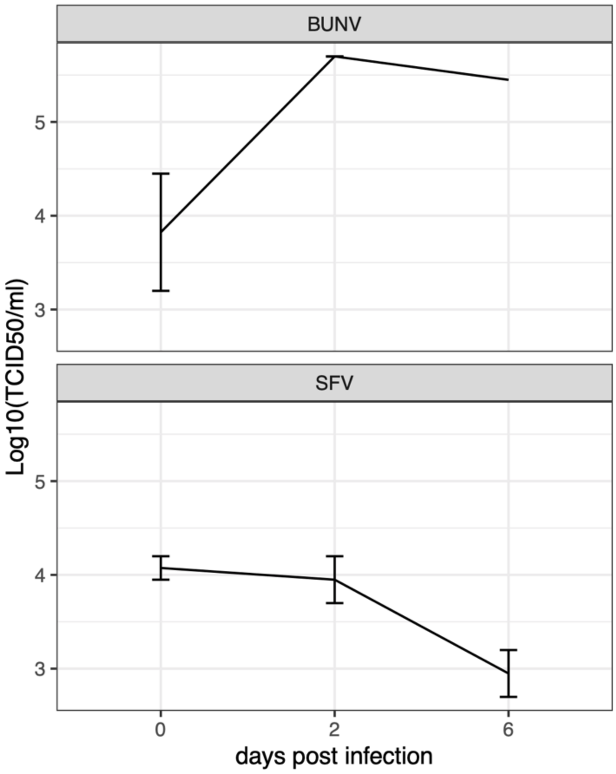

Figure S1. BUNV and SFV growth kinetics in *Cx. quinquefasciatus*-derived HSU cells. HSU cells were inoculated either with BUNV-Nluc (MOI 2) or with SFV-NLuc (MOI 10). Viral replication was measured as TCID50/mL; two independent experiments (except BUNV Nluc 6dpi, *n* = 1) performed in technical duplicates are shown with standard errors. Growth kinetics were measured at 0, 2 and 6 days post-infection.

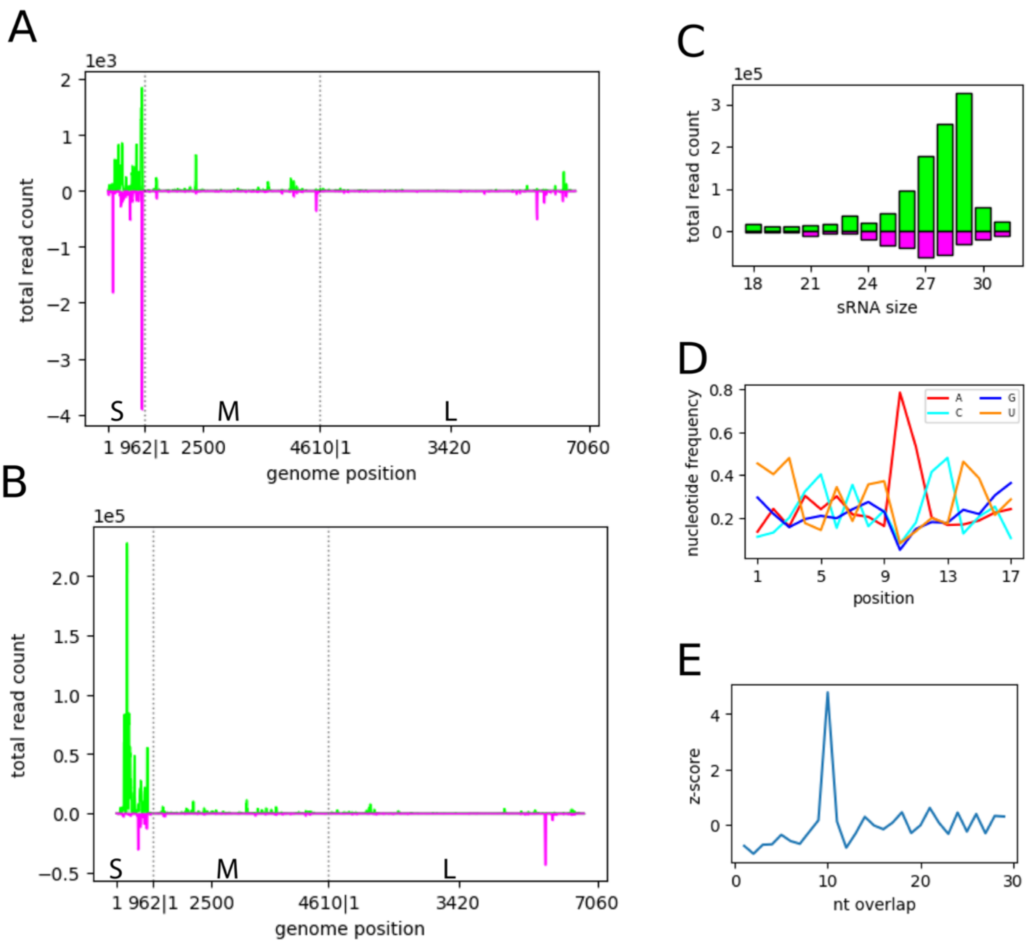

Figure S2. Second repeat of small RNA sequencing of BUNV-infected *Cx. quinquefasciatus*-derived HSU cells**.** Small RNAs of HSU cells were mapped to the BUNV genome and antigenome. (**A**) Distribution of the 21 nt small RNAs or (**B**) 26–29 nt small RNAs along the genome and antigenome of the three segments of BUNV (S, M, L). (**C**) Length distribution of BUNV-specific small RNAs. Positive numbers are RNAs mapping to the antigenome of BUNV (green), while negative numbers indicate RNAs mapping to the genome of BUNV (pink). Y-axis: absolute count of small RNAs. (**D**) Relative nucleotide frequency and conservation per position of 26–29 nt small RNAs mapping to the BUNV genome or antigenome. (**E**) The overlap z-score indicating the probability of overlap between the genome and antigenome of 26–29 nt BUNV-specific small RNAs was calculated. Two independent experiments were carried out, and the results of one experiment are shown here (see Figure 2).

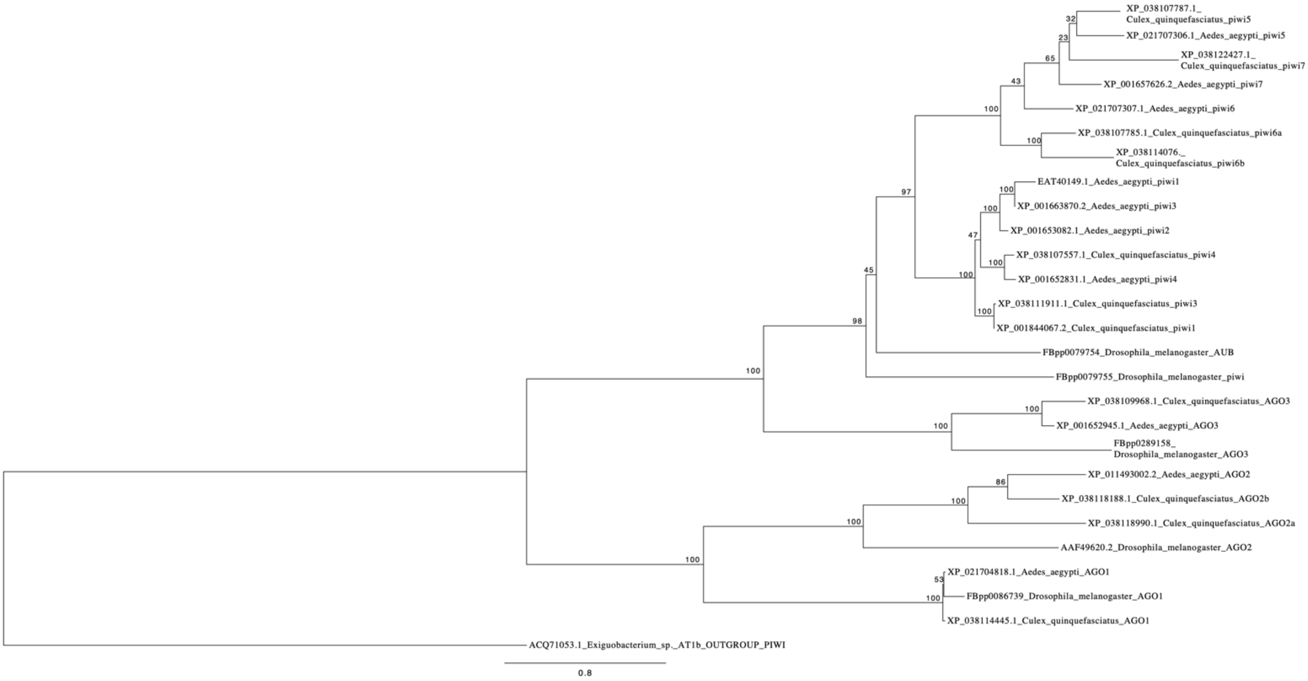

Figure S3. RNAi-related proteins in *Cx. quinquefasciatus*. RNAi-related Argonaute and PIWI proteins in *Cx. quinquefasciatus* were searched based on similarity to annotated *Ae. aegypti* proteins. Protein sequences from *Ae. aegypti*, *Cx. quinquefasciatus* and *D. melanogaster* were aligned, and a maximum-likelihood-based phylogenetic tree was built using Phyml, with 1000 bootstraps and *Exiguobacterium* sp. PIWI as an outgroup. Bootstrap values are given as percentages. Both *Cx. quinquefasciatus* and *Ae. aegypti* have seven Piwi proteins. However, Piwi5 and Piwi4 proteins of *Ae. aegypti* and *Cx. quinquefasciatus* clustered together, while other Piwis did not directly cluster with their *Ae. aegypti* counterparts and had a different common ancestor. For instance, *Ae. aegypti* Piwi1, 2 and 3 cluster closely together and come from a common ancestor, but *Cx. quinquefasciatus* has only two Piwis, named Piwi1 and 3, which constituted a separate cluster from *Ae. aegypti* Piwi1, 2 and 3. In addition, *Cx. quinquefasciatus* has two Piwi6 and two Ago2 homologues (here named Piwi 6a/6b and Ago2a/2b, respectively), whereas *Ae. aegypti* only encodes one of each.
